# Functional Group Chemistry Modulates Cellular Responses to Soluble Gelatin Derivatives Independent of Crosslinking

**DOI:** 10.3390/biom16060836

**Published:** 2026-06-05

**Authors:** Pekik Wiji Prasetyaningrum, Shinji Sakai

**Affiliations:** Division of Chemical Engineering, Department of Materials Engineering Science, Graduate School of Engineering Science, The University of Osaka, 1-3 Machikaneyama-cho, Toyonaka 560-8531, Osaka, Japan; pekikwiji@cheng.es.osaka-u.ac.jp

**Keywords:** gelatin, GelMA, GelNB, GelPH, cytocompatibility

## Abstract

Gelatin functionalized with methacrylate (GelMA), norbornene (GelNB), and phenol (GelPH) has been studied as a precursor for crosslinkable biomaterials in tissue engineering. Cytocompatibility is usually assessed post-hydrogel formation, but cells briefly encounter soluble precursors during preparation and residual functional groups that may remain post-gelation. However, the biological effects of these uncrosslinked derivatives remain poorly understood. This study systematically investigated the impact of functional group chemistry on cellular responses under uncrosslinking conditions. Four cell lines, including fibroblasts (BALB/3T3), cervical cancer cells (HeLa), mesenchymal stem cells (UE7T-13), and neuronal cells (PC-12), were exposed to soluble GelMA, GelNB, and GelPH at matched polymer concentrations and degrees of functionalization (~50%). The results showed no overt cytotoxicity. The detectable differences were subtle and strongly cell type-dependent. HeLa and PC-12 cells showed no clear differences from untreated controls in most parameters. BALB/3T3 fibroblasts showed mainly GelMA-associated differences in mitochondrial activity, proliferation, and morphology. In the UE7T-13 cells, GelMA-treated cells showed the largest deviations from controls across the readouts examined, including mitochondrial activity, cell area, phenotype-related gene expression, and some osteogenic markers, while GelPH was intermediate and GelNB was comparable to controls. These findings underscore the importance of considering residual precursor effects in gelatin-based biomaterial design.

## 1. Introduction

Gelatin-derived biomaterials have been extensively studied for tissue engineering applications, particularly as three-dimensional (3D) hydrogel matrices for cell culture, owing to their inherent biocompatibility, biodegradability, and resemblance to the native extracellular matrix (ECM) [1,2,3]. These materials also yield hydrogels with tunable mechanical properties and improved stability compared to native gelatin. Among the various recently developed functionalized derivatives, gelatin–methacrylate (GelMA), gelatin–norbornene (GelNB), and gelatin–phenol (GelPH) have been widely studied. These materials enable covalent crosslinking via free-radical polymerization, thiol-ene or inverse-electron-demand Diels–Alder click chemistry, and phenol coupling reactions, respectively [4,5,6,7]. Although these systems differ in their crosslinking strategies, the introduction of functional groups inevitably alters the physicochemical properties of the gelatin backbone, potentially influencing its interactions with cells [2,8].

The cytocompatibility of functionalized gelatin has been predominantly evaluated in crosslinked hydrogels and during crosslinking. In such systems, cellular responses are influenced by multiple factors, including photoinitiators, light exposure, enzymatic catalysts, crosslinking kinetics, and network architecture [9,10,11,12,13,14,15,16,17,18]. Consequently, cytocompatibility is typically assessed in the context of a full crosslinking process, implicitly assuming that the introduced functional groups are biologically inert once incorporated into the hydrogel network. However, in practical applications, cells are exposed to soluble uncrosslinked gelatin derivatives before and during gelation, and residual reactive moieties may persist after crosslinking [9,10,11,12,13,14,15,16,17,18]. These transient interactions raise the possibility that functional group chemistry may contribute to differences in cellular responses beyond the bulk hydrogel properties.

Göckler et al. [9] compared the precursor cytotoxicity of GelMA and GelNB at a high polymer concentration (5% *w*/*v*) and reported that uncrosslinked GelMA induced a marked reduction in cell viability within 2 h, whereas GelNB maintained higher cytocompatibility over the same period. This study provides initial evidence that functional group chemistry is associated with cellular responses under uncrosslinking conditions. However, the evaluation was limited to short-term viability endpoints and a single cell type, leaving several important questions unanswered. Furthermore, the high polymer concentration used reflects an overt exposure condition rather than the lower residual concentrations expected during or after construct fabrication. In biomedical applications, uncrosslinked materials may leach from partially crosslinked hydrogels and interact with surrounding tissues, potentially leading to systemic exposure depending on the route of administration [19,20,21,22,23,24,25]. Therefore, understanding cellular responses under such conditions is potentially relevant for the safety evaluation of gelatin derivative-based constructs in biomedical applications. Beyond this, no systematic comparative study has directly isolated the intrinsic effects of GelMA, GelNB, and GelPH on cellular behavior, independent of the crosslinking chemistry. Existing reports vary widely in terms of material concentration, degree of functionalization, crosslinking, and experimental conditions [9,10,16,26], making it difficult to determine whether the biological differences arise from functional group chemistry or downstream gelation processes. Consequently, it remains unclear whether methacrylate, norbornene, and phenolic modifications differentially influence cellular responses when present on uncrosslinked gelatin. To the best of our knowledge, no study has systematically isolated and compared the intrinsic biological effects of methacrylate, norbornene, and phenolic functionalization under matched conditions in the absence of crosslinking stimuli.

To address this gap, we hypothesized that functional group chemistry may intrinsically modulate cellular responses, independent of hydrogel formation. Accordingly, this study systematically compared the effects of methacrylate, norbornene, and phenolic functionalization on cell behavior under controlled, uncrosslinking conditions. To minimize the confounding effects, gelatin derivatives with comparable degrees of functionalization (~50%) were tested at identical polymer concentrations without crosslinking stimuli. Four cell lines with distinct biological characteristics—fibroblasts (BALB/3T3), cervical cancer cells (HeLa), mesenchymal stem cells (UE7T-13), and neuronal cells (PC-12)—were exposed to soluble gelatin derivatives, and cellular responses were evaluated using mitochondrial activity assays, proliferation analysis, morphological characterization, phenotype-related gene expression analysis, and osteogenic differentiation assays. This study provides a controlled comparative basis for evaluating residual soluble gelatin derivatives with different functionalization chemistries under the tested uncrosslinked conditions, supporting more informed selection of gelatin-based materials for biomedical applications.

## 2. Materials and Methods

### 2.1. Materials

Gelatin type A from porcine skin (approximately 300 g bloom) and methacrylic anhydride (approximately 94% purity) were purchased from Sigma-Aldrich (St. Louis, MO, USA). 5-norbornene-2,3-dicarboxylic anhydride (>97% purity) and 3-(4-hydroxyphenyl)propionic acid (HPPA) (>98% purity) were purchased from Tokyo Chemical Industry (Tokyo, Japan). N-Hydroxysuccinimide (NHS), 2,4,6-trinitrobenzenesulfonic acid (TNBS; 98% purity), and *N,N*-dimethylformamide (DMF) (95% purity) were purchased from Fujifilm Wako Pure Chemicals (Osaka, Japan). 1-Ethyl-3-(3-dimethylaminopropyl) carbodiimide (EDC), tyramine hydrochloride, and cell counting reagent (Cell Counting Kit 8) were purchased from the Peptide Institute (Osaka, Japan), Chem-Impex (Wood Dale, IL, USA), and Dojindo (Kumamoto, Japan), respectively. Dulbecco’s modified Eagle’s medium (DMEM) was purchased from Nissui Pharmaceutical Co., Ltd. (Tokyo, Japan). RPMI-1640 medium was purchased from Shimadzu Diagnostics (Kyoto, Japan). Osteoblast differentiation medium was purchased from Cell Applications (San Diego, CA, USA). The reagents for RNA isolation (RNA isolation kit) and cDNA synthesis (PrimeScript RT Master Mix) were purchased from Takara Bio (Shiga, Japan).

### 2.2. Synthesis and Characterization of Functionalized Gelatin Derivatives

Gelatin methacrylation was performed by dissolving 1 g of gelatin in 10 mL of phosphate-buffered saline (PBS, pH 7.4). Subsequently, 0.5 mL of methacrylic anhydride was added to the gelatin solution, and the mixture was reacted for 1 h at 50 °C. The reaction was quenched by adding PBS to four times the original reaction volume [27].

Norbornene groups were introduced into the gelatin backbone by reacting 1 g of gelatin with 1 g of 5-norbornene-2,3-dicarboxylic anhydride in 10 mL PBS. The reaction was performed at 40 °C for 36 h, and the pH was maintained at 8 by adjusting it every 12 h. The reaction was terminated by adding PBS thrice to the reaction volume [10].

Phenolic gelatin was synthesized by activating HPPA. Briefly, HPPA (2.64 g) was mixed with EDC (1.14 g) and NHS (0.96 g) in 50 mL of DMF buffer (pH 4.7), and the mixture was stirred for 5 h. The gelatin solution was prepared by dissolving 4 g of gelatin in 50 mL of DMF buffer. The gelatin solution was then mixed with the activated HPPA solution and reacted for 20 h at 37 °C [28,29].

The synthesized gelatin derivatives were purified by dialysis against distilled water using 12–14 kDa MWCO tubing at 40 °C for 3–5 days, with the dialysate replaced at least twice daily. For GelPH, removal of unreacted HPPA was confirmed by monitoring the dialysate absorbance at 275 nm until no detectable peak was observed (Figure S1). For GelMA and GelNB, dialysis was monitored by measuring the total dissolved solids (TDS) of the dialysate to assess the removal of dissolved salts and low-molecular-weight by-products generated during the reactions. Dialysis continued until the TDS value approached 0 ppm. The purified polymers were subsequently lyophilized. The gelatin derivative products were then stored at 4 °C. Characterization was performed using ^1^H NMR (JNM ECS-400; JEOL, Tokyo, Japan), and the degree of functionalization was quantified using the TNBS assay.

### 2.3. Cell Culture

Mouse fibroblasts (BALB/3T3), human cervical cancer cells (HeLa), immortalized human bone marrow–derived mesenchymal stem cells (UE7T-13), and rat pheochromocytoma neuronal cells (PC-12) were obtained from RIKEN Cell Bank (Ibaraki, Japan). BALB/3T3, UE7T-13, and HeLa cells were cultured in DMEM supplemented with 10% *v*/*v* fetal bovine serum (FBS), whereas PC-12 cells were cultured in RPMI-1640 medium supplemented with 10% *v*/*v* horse serum and 5% *v*/*v* FBS. All cells were maintained in a humidified incubator at 37 °C with 5% CO_2_.

### 2.4. Treatment with Functionalized Gelatin Derivatives

Prior to cell exposure, native gelatin and functionalized gelatin derivatives were sterilized with ethanol. Briefly, the lyophilized polymers were sprayed with a sufficient volume of 70% *v*/*v* ethanol under aseptic conditions, then dried under vacuum for 2 days to evaporate the residual ethanol. After sterilization, the polymers were dissolved in culture medium and used to treat cells at a final concentration of 0.1% *w*/*v*. Control cells were cultured under identical conditions without polymer additives. Unless otherwise specified, treatment was initiated 48 h after cell seeding and maintained throughout each experiment. All reported time points in each assay refer to the time elapsed after the initiation of gelatin derivative treatment, rather than the time after cell seeding.

### 2.5. Mitochondrial Activity Assay

The mitochondrial activity of the treated cells was measured at defined growth phases (lag, exponential, and stationary phases) using Cell Counting Kit-8 at 10% *v*/*v* (2 h incubation). Absorbance was measured at 450 nm using a plate reader (SpectraMax iD3, Molecular Devices, San Jose, CA, USA). The mitochondrial activity per cell was calculated by normalizing the absorbance values to the total number of cells.

### 2.6. Cell Proliferation Analysis

The proliferation profile of treated cells was continuously monitored using a live-cell imaging system (CM20; Olympus, Tokyo, Japan) until the cells reached stationary phase. Cell growth curves were generated by plotting cell numbers against time. The doubling time was calculated from the exponential growth phase (R^2^ > 0.95) and was determined independently for each cell type based on the observed growth kinetics.

### 2.7. Cell Morphology Analysis

The effects of functionalized gelatin treatment on cell morphology were assessed by capturing micrographs at defined growth phases (lag, exponential, and stationary phases), with sampling time points adjusted for each cell type to account for differences in growth kinetics. The cell area and circularity were quantified after image segmentation using the Cellpose web platform (Cellpose: Huggingface.co/spaces/mouseland/cellpose). The segmentation outputs were subsequently analyzed using the image analysis software (ImageJ 1.52a, NIH, Bethesda, MD, USA).

### 2.8. Gene Expression Analysis (RT-qPCR)

UE7T-13 cells were treated with the functionalized gelatin derivatives for 3 days. The expression levels of Vimentin, GSTP1, SOX2, and Endoglin were evaluated. Total RNA was reverse-transcribed using the PrimeScript RT Master Mix (Takara Bio). qPCR was performed using the TB Green Master Mix (Takara Bio), with GAPDH as the housekeeping gene. Expression levels were normalized to untreated cells using the 2^−ΔΔCt^ method.

### 2.9. Osteogenic Differentiation

UE7T-13 cells were treated with functionalized gelatin derivatives. After reaching approximately 80% confluence, osteogenic differentiation was induced by replacing the culture medium with osteogenic differentiation medium. Differentiation was evaluated by Alizarin Red staining on day 14, and the staining intensity was quantified using image analysis software (ImageJ (version 1.52a), NIH, Bethesda, MD, USA). Osteogenic marker gene expression (COL1A1, RUNX2, OCN) was analyzed on day 20 by RT-qPCR, with expression levels normalized to undifferentiated cells using the 2^−ΔΔCt^ method.

### 2.10. Statistical Analysis

Data were analyzed using spreadsheet software (Excel 2024 (version 2605 Build 16.0.20026.20076), Microsoft, Redmond, WA, USA) and statistical analysis and graphing software (OriginPro 2026 (version 10.3.0.180), OriginLab, Northampton, MA, USA). The Grubbs’ test was used to identify outliers. Data are expressed as mean ± SD. Statistical significance was determined using one-way analysis of variance (ANOVA) followed by Tukey’s HSD post hoc test, with *n* = 3, unless otherwise indicated.

## 3. Results

### 3.1. Functionalization of Gelatin

The successful modification of gelatin was confirmed by ^1^H NMR analysis (Figure S2). GelMA was identified by the presence of two characteristic peaks at 5.4–5.7 ppm, corresponding to the vinyl protons of the methacrylate groups [30]. The alkene protons of the norbornene groups in GelNB were verified by a distinct peak at 6.1 ppm [31]. The introduction of phenolic functionalities in GelPH was indicated by two peaks at approximately 6.8 and 7.1 ppm, corresponding to the aromatic protons of the phenol groups [18,28,29]. Quantification using the TNBS assay indicated that native gelatin contained 0.31 mmol/g of primary amines, whereas GelMA, GelNB, and GelPH exhibited residual amine contents of 0.17, 0.17, and 0.15 mmol/g, corresponding to degrees of functionalization (DoF) of 55%, 56%, and 50%, respectively. These results confirmed the successful and comparable functionalization across all derivatives, enabling a direct comparison of their biological effects.

### 3.2. Effects of Functionalized Gelatin Derivatives on Metabolic Activity, Proliferation, and Morphology

Cellular responses to the functionalized gelatin derivatives (GelMA, GelPH, and GelNB) were evaluated using multiple cell types with distinct biological characteristics, including fibroblasts (BALB/3T3), cervical cancer cells (HeLa), mesenchymal stem cells (UE7T-13), and neuronal cells (PC-12). Multiple cell types were used to allow for a broader assessment of the cytocompatibility of the modified gelatin polymers. Cellular responses were assessed based on mitochondrial activity, proliferation, and morphological analyses. All gelatin derivatives were prepared with similar degrees of functionalization (~50%) and tested at a fixed concentration of 0.1% *w*/*v* across all groups. This design was used to facilitate the comparison of GelMA, GelNB, and GelPH under uncrosslinking conditions.

#### 3.2.1. BALB/3T3 Cells

**Figure 1:**
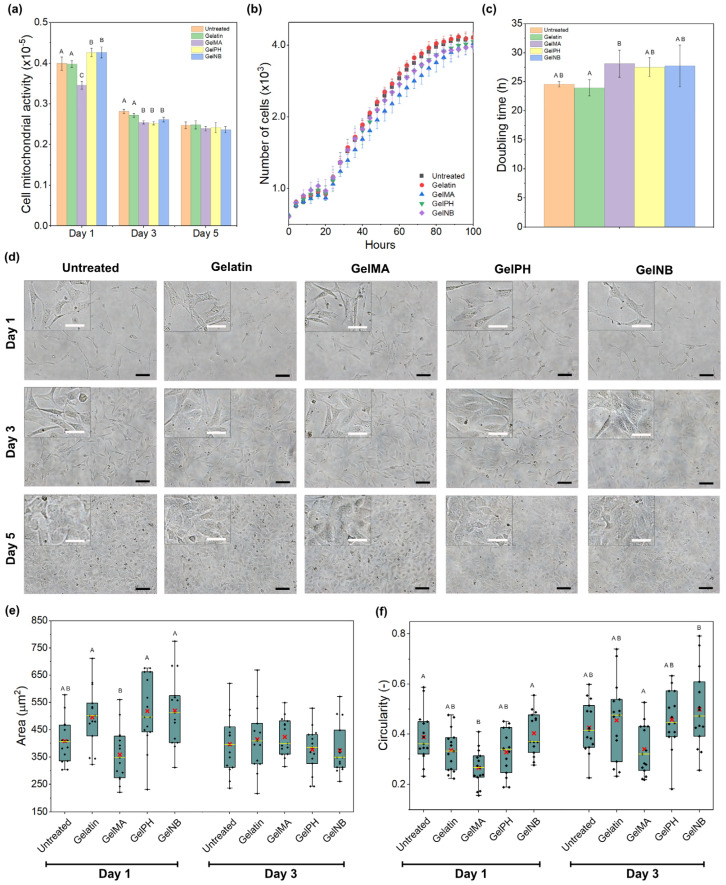
Cellular responses of BALB/3T3 cells following treatment with soluble gelatin derivatives. (**a**) mitochondrial activity normalized per cell (*n* = 4), (**b**) cell growth profiles (*n* = 5), (**c**) doubling time (*n* = 5), (**d**) representative phase-contrast images acquired on days 1, 3, and 5 after treatment, and quantitative analysis of (**e**) cell area and (**f**) circularity on days 1 and 3 (*n* = 15). Scale bars: black, 100 µm; white, 50 µm. In the box plots, the red cross represents the mean, and the yellow line represents the median. Data represent mean ± SD. Groups not sharing the same letter are significantly different (*p* < 0.05, one-way ANOVA with Tukey’s HSD). Groups sharing at least one letter are not significantly different. Groups without letters are not significantly different.

As shown in Figure 1a, the mitochondrial activity of BALB/3T3 cells on day 1 differed significantly between groups. GelMA-treated cells exhibited significantly lower mitochondrial activity than the other groups (*p* < 0.05, Tukey’s HSD). In contrast, GelPH- and GelNB-treated cells displayed higher values (*p* < 0.05, Tukey’s HSD), whereas native gelatin-treated cells showed no significant differences compared to the untreated control. By day 3, mitochondrial activity had decreased in all groups treated with the gelatin derivatives (*p* < 0.05, Tukey’s HSD). On day 5, mitochondrial activity was comparable across all groups, with no significant differences. This may be due to the high cell confluency reached at this time point, at which mitochondrial activity tended to plateau because of space and nutrient limitations [32,33].

The cell proliferation analysis exhibited similar growth profiles among all groups throughout the culture period (Figure 1b). However, minor differences in cell numbers were observed, with BALB/3T3 cells treated with functionalized gelatin derivatives showing slightly fewer cells than those treated with native gelatin or untreated groups. Consistent with this observation, the calculated doubling time was slightly longer in the derivative-treated groups (Figure 1c). Post hoc analysis revealed that GelMA-treated cells had a significantly longer doubling time than native gelatin-treated cells (*p* < 0.05, Tukey’s HSD), while untreated, GelPH-, and GelNB-treated cells had doubling times that were not significantly different from either native gelatin or GelMA-treated cells (Figure 1c).

Phase-contrast microscopy showed that BALB/3T3 cells maintained a typical fibroblast-like morphology [34] under all treatment conditions and progressively increased in confluence from day 1 to 5 (Figure 1d). However, GelMA-treated cells appeared slightly thinner than those in the other groups. This was supported by quantitative morphological analysis, in which the GelMA-treated cells had a significantly smaller cell area than the native gelatin group on day 1 (*p* < 0.05, Tukey’s HSD), whereas no significant differences were observed among the other groups (Figure 1e). Circularity analysis similarly revealed significantly lower values in GelMA-treated cells than in the untreated controls (*p* < 0.05, Tukey’s HSD), whereas the other groups showed no significant differences (Figure 1f). By day 3, the circularity remained lower in the GelMA group, whereas the cell area was comparable across all groups (Figure 1f). Collectively, these findings indicated that GelMA had the most noticeable effect on cell morphology, particularly at early time points, whereas the other derivatives appeared to have minimal effects.

Overall, these results demonstrate that gelatin and its derivatives do not exhibit strong cytotoxic effects in BALB/3T3 cells. However, the gelatin derivatives induced modest changes in cellular responses, particularly in the GelMA-treated group, which exhibited lower mitochondrial activity, longer doubling times, and thinner cell morphology than other groups.

#### 3.2.2. HeLa Cells

**Figure 2:**
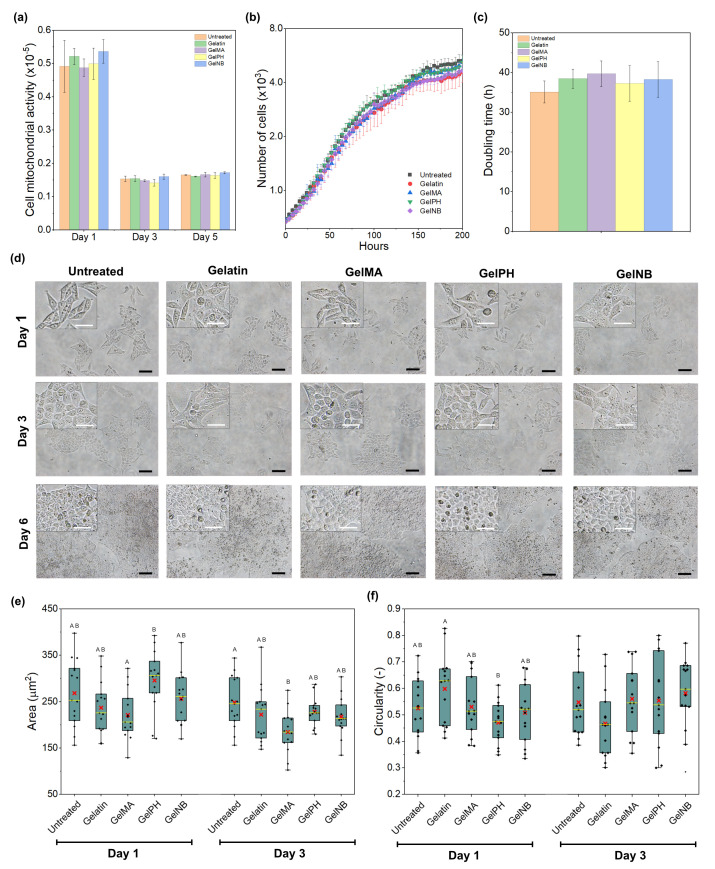
Cellular responses of HeLa cells following treatment with soluble gelatin derivatives. (**a**) mitochondrial activity normalized per cell (*n* = 4), (**b**) cell growth profiles (*n* = 5), (**c**) doubling time (*n* = 5), (**d**) representative phase-contrast images acquired on days 1, 3, and 6 after treatment, and quantitative analysis of (**e**) cell area and (**f**) circularity on days 1 and 3 (*n* = 15). Scale bars: black, 100 µm; white, 50 µm. In the box plots, the red cross represents the mean, and the yellow line represents the median. Data represent mean ± SD. Groups not sharing the same letter are significantly different (*p* < 0.05, one-way ANOVA with Tukey’s HSD). Groups sharing at least one letter are not significantly different. Groups without letters are not significantly different.

HeLa cells showed comparable mitochondrial activity across all groups at each time point (Figure 2a), with no significant differences observed. As observed in BALB/3T3 cells, the mitochondrial activity in HeLa cells gradually decreased over time. Consistent with the mitochondrial activity data, cell proliferation analysis revealed similar growth profiles across all groups throughout the culture period (Figure 2b), and no significant differences were observed in the calculated doubling times (Figure 2c).

Phase-contrast microscopy showed that HeLa cells maintained their typical epithelial-like morphology under all treatment conditions and progressively increased in confluence over the 6-day observation period, with no apparent morphological abnormalities in any group (Figure 2d) [35]. Quantitative morphological analysis showed that on day 1, GelMA-treated cells had a significantly smaller cell area, whereas GelPH-treated cells had a significantly larger cell area than the corresponding comparison groups (*p* < 0.05, Tukey’s HSD) (Figure 2e). The circularity values were lower in the GelPH-treated group on day 1 (Figure 2f). By day 3, GelMA-treated cells exhibited a significantly smaller cell area than native gelatin-treated cells (*p* < 0.05, Tukey’s HSD). Circularity was comparable across all groups on day 3, with no significant differences detected.

Collectively, these results implied that gelatin and its functionalized derivatives did not induce noticeable effects on cellular metabolic activity or proliferation. However, a subtle effect on cell morphology was observed in the GelMA-treated cells, which tended to have smaller cell areas than the other groups.

#### 3.2.3. UE7T-13 Cells

**Figure 3:**
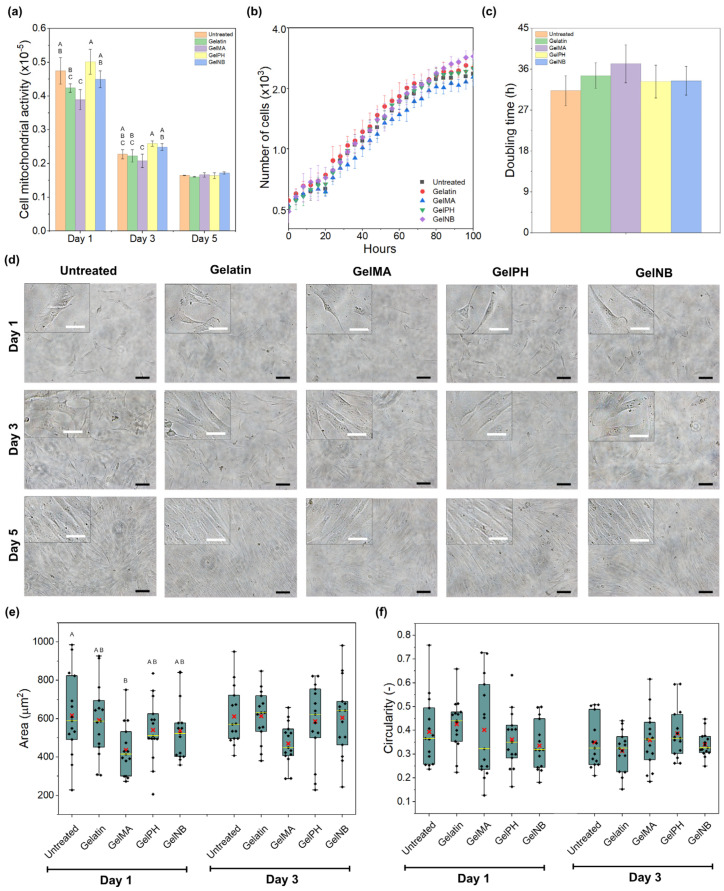
Cellular responses of UE7T-13 cells following treatment with soluble gelatin derivatives. (**a**) mitochondrial activity normalized per cell (*n* = 4), (**b**) cell growth profiles (*n* = 5), (**c**) doubling time (*n* = 5), (**d**) representative phase-contrast images acquired on days 1, 3, and 5 after treatment, and quantitative analysis of (**e**) cell area and (**f**) circularity on days 1 and 3 (*n* = 15). Scale bars: black, 100 µm; white, 50 µm. In the box plots, the red cross represents the mean, and the yellow line represents the median. Data represent mean ± SD. Groups not sharing the same letter are significantly different (*p* < 0.05, one-way ANOVA with Tukey’s HSD). Groups sharing at least one letter are not significantly different. Groups without letters are not significantly different.

The mitochondrial activity of UE7T-13 cells varied with time and treatment conditions (Figure 3a). On day 1, GelMA-treated cells exhibited significantly lower mitochondrial activity than the untreated controls (*p* < 0.05, Tukey’s HSD), whereas gelatin-, GelPH-, and GelNB-treated cells did not differ significantly from the untreated controls. By day 3, the mitochondrial activity was comparable across all groups, and no statistically significant differences were observed between any treatment and the untreated control. At later time points, the mitochondrial activity was similar across all groups. As observed in other cells, the mitochondrial activity gradually decreased over time.

Cell proliferation analysis showed that UE7T-13 cells in all groups exhibited similar growth trends during the observation period (Figure 3b). Although GelMA-treated groups tended to show lower cell numbers than the untreated group, the overall proliferation profiles remained comparable among the groups. Consistent with this, doubling time analysis showed no statistically significant differences among the treatment groups (Figure 3c).

Phase-contrast microscopy revealed that UE7T-13 cells retained their typical spindle-shaped mesenchymal morphology [36] under all treatment conditions and gradually increased in confluence during the culture period (Figure 3d). Quantitative analysis of the cell area showed that the GelMA-treated cells had a significantly smaller area than the untreated cells on day 1. On day 3, GelMA-treated cells tended to have smaller cell areas; however, no statistically significant differences were observed between the groups (Figure 3e). Circularity analysis revealed no statistically significant differences between groups at either time point (Figure 3f).

These findings suggest that UE7T-13 cells generally tolerate gelatin and its functionalized derivatives. However, GelMA treatment was associated with slightly lower early mitochondrial activity and reduced cell area compared with the untreated control.

#### 3.2.4. PC-12 Cells

**Figure 4:**
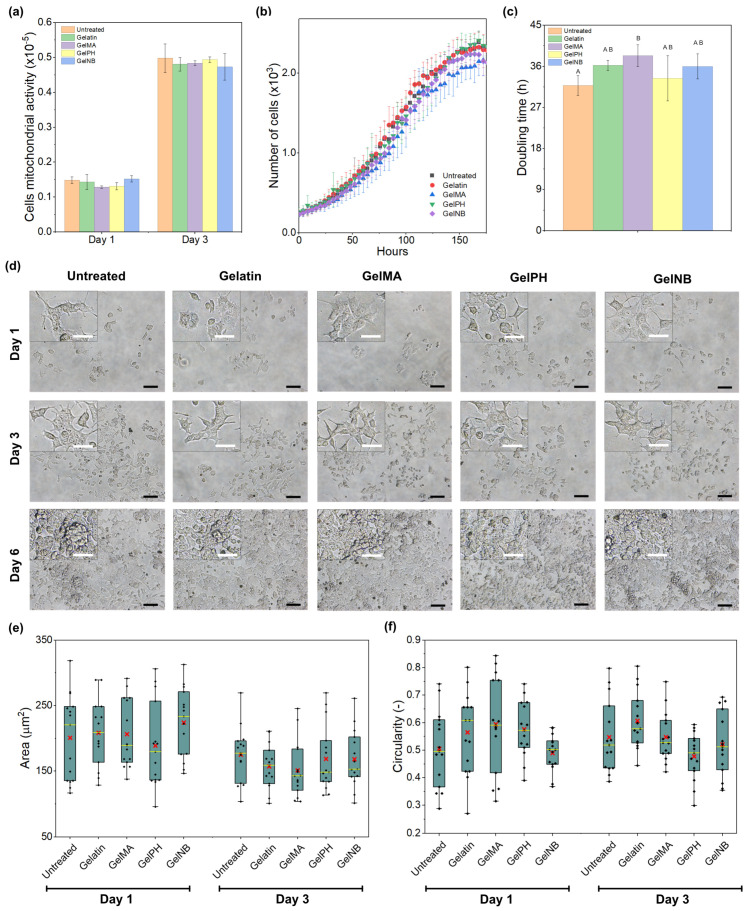
Cellular responses of PC-12 cells following treatment with soluble gelatin derivatives. (**a**) mitochondrial activity normalized per cell (*n* = 4), (**b**) cell growth profiles (*n* = 5), (**c**) doubling time (*n* = 5), (**d**) representative phase-contrast images acquired on days 1, 3, and 6 after treatment, and quantitative analysis of (**e**) cell area and (**f**) circularity on days 1 and 3 (*n* = 15). Scale bars: black, 100 µm; white, 50 µm. In the box plots, the red cross represents the mean, and the yellow line represents the median. Data represent mean ± SD. Groups not sharing the same letter are significantly different (*p* < 0.05, one-way ANOVA with Tukey’s HSD). Groups sharing at least one letter are not significantly different. Groups without letters are not significantly different.

As shown in Figure 4a, the mitochondrial activity of PC-12 cells was similar across all treatment groups on day 1, with no apparent differences compared to the untreated control. By day 3, mitochondrial activity had increased in all groups and remained broadly comparable among treatments, possibly reflecting metabolic stabilization following the initial attachment period [37]. Day 6 data were excluded because extensive cell detachment occurred at high confluency, compromising the accuracy of the per-cell mitochondrial activity calculations.

PC-12 cells exhibited similar proliferation patterns across all groups during the observation period (Figure 4b). Nevertheless, the GelMA-treated group consistently displayed lower cell numbers than the untreated control, and doubling time analysis revealed a significantly longer doubling time in GelMA-treated cells (*p* < 0.05, Tukey’s HSD), whereas the other groups showed comparable values to untreated cells (Figure 4c).

PC-12 cells maintained their characteristic round and clustered morphology [38] under all treatment conditions and progressively increased in confluence during the incubation period (Figure 4d). Quantitative morphological analysis revealed similar cell areas and circularity values across all groups (Figure 4e,f). Overall, PC-12 cells showed similar responses across groups, although GelMA-treated cells tended to have lower proliferation than untreated cells.

### 3.3. Gene Expression Responses in UE7T-13 Cells

**Figure 5:**
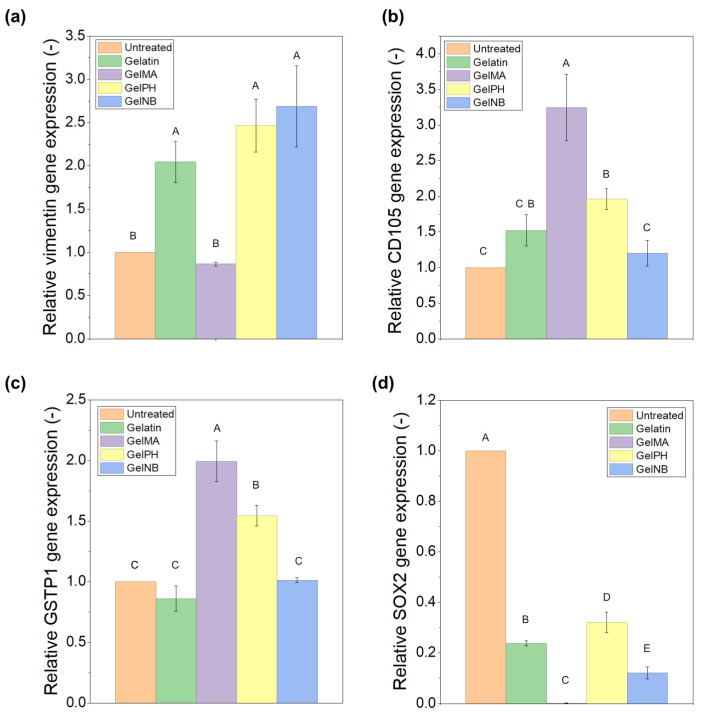
Gene expression responses of UE7T-13 cells after 3 days of treatment with soluble gelatin derivatives. Relative gene expression of (**a**) vimentin, (**b**) endoglin (CD105), (**c**) GSTP1, and (**d**) SOX2, normalized to the housekeeping gene GAPDH and presented relative to untreated cells. Data represent mean ± SD (*n* = 3). Groups not sharing the same letter are significantly different (*p* < 0.05, one-way ANOVA with Tukey’s HSD). Groups sharing at least one letter are not significantly different.

To further evaluate whether exposure to functionalized gelatin derivatives was associated with changes in gene expression in UE7T-13 cells, markers related to mesenchymal identity, stemness, and cellular stress responses were analyzed. The relative expression of the four genes was analyzed after 3 days of treatment (Figure 5). The genes analyzed were vimentin, a canonical mesenchymal marker involved in cytoskeletal organization [39,40,41]; endoglin (CD105), a TGF-β co-receptor and surface marker of mesenchymal stromal cells [42]; SOX2, a transcription factor associated with stemness and self-renewal [43]; and GSTP1, a detoxification enzyme indicative of oxidative stress responses [44].

Vimentin expression differed among the treatment groups (Figure 5a). GelMA-treated cells showed a slightly lower mean expression level than untreated control cells, although this difference was not statistically significant. In contrast, native gelatin-, GelPH-, and GelNB-treated cells exhibited significantly higher vimentin expression than the untreated control cells (*p* < 0.05, Tukey’s HSD).

Endoglin (CD105) expression differed among the treatment groups (Figure 5b). GelMA-treated cells exhibited the highest expression level, significantly higher than those in all other groups (*p* < 0.05, Tukey’s HSD). GelPH-treated cells showed intermediate expression levels compared to the untreated control, native gelatin, and GelNB groups (*p* < 0.05, Tukey’s HSD). The untreated control, native gelatin, and GelNB groups exhibited comparable expression levels.

GSTP1 expression also varied among the treatments (Figure 5c). GelMA-treated cells showed the highest expression level, significantly higher than those in all other groups (*p* < 0.05, Tukey’s HSD). GelPH-treated cells exhibited intermediate expression, which was significantly higher than that of the untreated control, native gelatin, and GelNB groups (*p* < 0.05, Tukey’s HSD). Native gelatin- and GelNB-treated cells showed expression levels comparable to those of the untreated control.

In contrast, all treatment groups had lower SOX2 expression levels than the untreated controls (Figure 5d). Among the derivatives, GelMA-treated cells showed the lowest SOX2 expression, followed by GelNB, native gelatin, and GelPH; all groups were significantly different from one another (*p* < 0.05, Tukey’s HSD).

### 3.4. Osteogenic Differentiation of UE7T-13 Cells Treated with Gelatin Derivatives

Gelatin derivatives have been widely studied as substrates for 3D cell encapsulation in biomedical applications [1,2,3]. Therefore, it is important to evaluate whether exposure to uncrosslinked gelatin derivatives affects the cellular functions. In this study, the osteogenic differentiation potential of UE7T-13 cells treated with gelatin derivatives was evaluated and characterized using Alizarin Red staining and osteogenic marker gene expression analysis.

**Figure 6:**
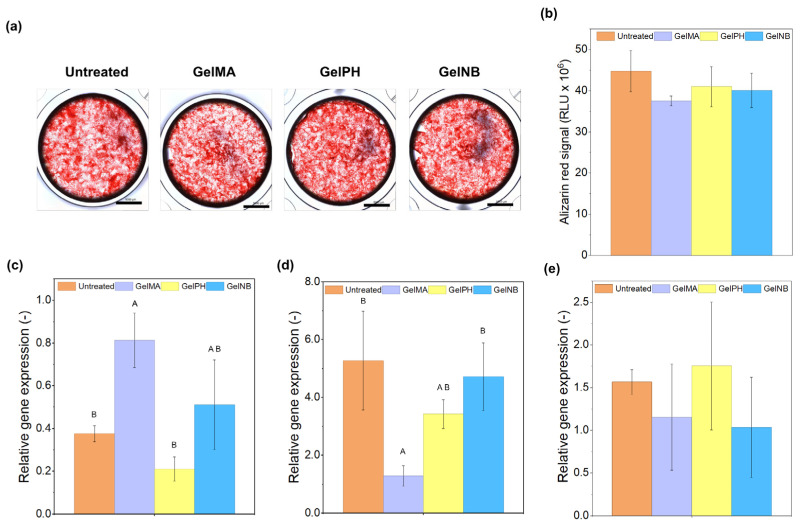
Osteogenic differentiation of gelatin derivative–treated UE7T-13 cells. (**a**) Representative Alizarin Red staining images and (**b**) quantified Alizarin Red staining intensity after 14 days of osteogenic induction, showing mineralized matrix formation. Relative expression levels of osteogenic marker genes after 20 days of differentiation, including (**c**) COL1A1, (**d**) RUNX2, and (**e**) osteocalcin (OCN), determined by RT-qPCR. Gene expression levels were normalized to GAPDH and expressed relative to undifferentiated cells. Scale bars: 5 mm. Data represent mean ± SD (*n* = 3). Groups not sharing the same letter are significantly different (*p* < 0.05, one-way ANOVA with Tukey’s HSD). Groups without letters are not significantly different.

All groups exhibited positive Alizarin Red staining after 14 days of osteogenic induction, which was consistent with mineralized matrix formation (Figure 6a). Although mineralized matrix formation was observed in all groups, the GelMA-treated group appeared to have slightly weaker staining intensity (Figure 6b).

As shown in Figure 6c, COL1A1 expression in all groups was lower than that in undifferentiated cells, including the untreated group. RUNX2 expression differed among the treatments (Figure 6d). GelMA-treated cells displayed the lowest expression and were significantly lower than the untreated and GelNB-treated groups (*p* < 0.05, Tukey’s HSD), whereas GelPH-treated cells showed intermediate expression (*p* < 0.05, Tukey’s HSD). The untreated and GelNB-treated groups exhibited comparable RUNX2 expression with no significant differences. In addition, OCN expression showed no clear differences among groups (Figure 6e).

## 4. Discussion

Gelatin derivatives, such as GelMA, GelNB, and GelPH, have been extensively investigated as hydrogel precursors for three-dimensional (3D) cell culture and tissue engineering because of their tunable crosslinking mechanisms and favorable biological properties [4,5,6,7]. Most previous studies have focused on how crosslinking-related factors and construct properties influence cellular responses [9,10,11,12,13,14,15,16,17,18]. In practical applications, cells are frequently exposed to modified gelatin before or during gelation, and residual functional groups may remain accessible when the crosslinking reactions are incomplete [9,45,46]. Therefore, understanding the potential biological effects of gelatin functionalization is important for evaluating the overall cytocompatibility of these materials.

The polymer concentration and degree of functionalization (DoF) were selected to reflect the biological scenario motivating this study. Gelatin derivatives are typically used at higher concentrations for hydrogel formation. However, after crosslinking, small amounts of unreacted functional groups may remain and interact with encapsulated cells. Moreover, in biomedical applications, uncrosslinked polymer chains may leach from the construct, and hydrogel degradation may release soluble fragments that still carry functional groups, allowing interaction with cells in the surrounding tissue [24,47,48]. Therefore, a concentration of 0.1% *w*/*v* was selected to approximate this low-exposure condition. This concentration also falls within the range commonly used for in vitro cytocompatibility testing of soluble polymers [49,50]. A DoF of approximately 50% was selected because this range has been reported to provide a practical balance between cytocompatibility and hydrogel mechanical properties [9,10,16,21,51]. At lower DoF, crosslinking efficiency and hydrogel stability may decrease due to limited functional groups. In contrast, excessive functionalization may consume a larger fraction of the gelatin backbone, including bioactive motifs that contribute to cell–material interactions. We recognize that cellular responses may depend on both polymer concentration and DoF [9,10]. Therefore, the present findings should be interpreted within the selected concentration and DoF range. Nevertheless, matching all three derivatives at a single, biologically relevant, and commonly used condition enabled direct comparison of GelMA, GelNB, and GelPH under uncrosslinking conditions.

Overall, the results indicate that the tested gelatin derivatives exhibited broadly acceptable cytocompatibility under the conditions examined. BALB/3T3, HeLa, UE7T-13, and PC-12 cells maintained normal metabolic activity, proliferation, and morphological characteristics when cultured in the presence of the soluble modified gelatin polymers. These results indicated that the introduction of methacrylate, phenolic, or norbornene groups did not cause pronounced cytotoxic effects in the soluble form under the tested conditions. Moreover, the absence of overt cytotoxicity suggests that any contribution from residual synthesis reagents was effectively minimized [50], indicating that the gelatin derivatives used in this study were adequately purified. These observations are consistent with the reported biocompatibility of gelatin-based biomaterials and suggest that moderate chemical functionalization does not substantially compromise cellular compatibility [5,6,52].

Despite their overall favorable cytocompatibility, small differences in cellular responses were observed between the gelatin derivatives and untreated cells. GelMA-treated cells exhibited relatively large differences, including reduced early mitochondrial activity, slower proliferation, and modest morphological changes in some cell types. GelPH was associated with intermediate responses, whereas GelNB responses were comparable to those of untreated cells. These findings are consistent with the study conducted by Göckler et al. [9], which showed that cells exposed to GelMA precursors had lower viability than those exposed to GelNB precursors.

Moreover, these cell types displayed distinct responses to the gelatin derivatives. Under the test conditions, BALB/3T3-treated cells exhibited the largest deviations from the control across the selected readouts. UE7T-13-treated cells showed noticeable differences in cellular responses, particularly in early mitochondrial activity and cell area. HeLa- and PC-12-treated cells exhibited minimal differences compared to untreated controls. One possible explanation is that differences in cell–matrix interaction dependence among cell types contribute to their differential sensitivity, as BALB/3T3 and UE7T-13 cells are generally considered to rely more strongly on extracellular matrix interactions [53,54,55,56]. PC-12 cells show moderate sensitivity, likely due to their neuroendocrine tumor origin, which may reduce their responsiveness to extracellular disturbances [57]. Moreover, the relatively limited response of HeLa cells is consistent with previous reports showing that some cancer-derived cell lines are less sensitive to microenvironmental changes [58]. However, these possibilities were not directly examined in this study. Overall, these results suggest that cellular responses to soluble gelatin derivatives may vary across cell types.

Gene expression analysis further revealed that gelatin functionalization might be associated with changes in gene expression in UE7T-13 cells, with GelMA showing the most pronounced difference. The modest reduction in vimentin expression in the GelMA group coincided with the decrease in cell area observed in the morphological analysis, consistent with a possible association between gene expression and cell morphology [59,60]. In contrast, vimentin expression was higher in the gelatin-, GelPH-, and GelNB-treated groups than in the untreated control group, although this trend was not accompanied by a clear increase in cell area. Therefore, the biological significance of these differences remains unclear. Additionally, endoglin expression varied across treatment groups, with the highest level observed in GelMA-treated cells, followed by GelPH-treated cells, whereas native gelatin- and GelNB-treated cells displayed levels comparable to those in the untreated groups. This pattern represents a derivative-dependent difference in phenotype-related gene expression at the single time point examined [42,61,62,63]. However, the functional significance of the altered endoglin expression was not examined in the present study. Furthermore, GSTP1 expression was elevated in GelMA- and GelPH-treated cells compared with that in the other groups, a pattern that may be consistent with a mild stress-related response, although stress pathway activation was not directly assessed in the present study [64,65]. In contrast, GelNB- and native gelatin-treated cells exhibited GSTP1 expression comparable to that of the untreated controls, suggesting that the differences in GSTP1 expression may reflect derivative-dependent differences rather than a response to the shared gelatin backbone. Additionally, all treatment groups showed a modest reduction in SOX2 expression, with the GelMA-treated cells showing the lowest level. The consistent pattern across all derivatives, regardless of the functional group identity, may indicate a shared response to the collagen-derived gelatin backbone rather than to any specific functional group chemistry [66]. These gene expression results were obtained from a single time point (day 3) with three biological replicates per group and should therefore be interpreted as preliminary observations of derivative-associated differences rather than as definitive characterization of the underlying regulatory dynamics. Time-course studies with larger sample sizes will be needed to determine whether these differences reflect transient responses, sustained reprogramming, or downstream functional consequences.

To evaluate whether the functionalized gelatin derivatives were associated with changes in cellular function, UE7T-13 cells were subjected to osteogenic differentiation following treatment with each derivative. All treatment groups showed mineralized matrix formation after induction, as evidenced by Alizarin Red staining, suggesting that osteogenic differentiation was maintained under the tested conditions. COL1A1 expression was lower in all groups than in undifferentiated cells, including the untreated control, which may reflect relatively high baseline COL1A1 expression in UE7T-13 cells prior to osteogenic induction [36,67]. Among the treated groups, GelNB-treated cells showed Alizarin Red staining, RUNX2 expression, and OCN expression comparable to those of the untreated controls. The GelPH-treated cells exhibited an intermediate response. Although GelMA-treated cells showed slightly weaker Alizarin Red staining, significantly lower RUNX2 expression, and higher COL1A1 expression than the untreated group, OCN expression did not differ between any treatment groups, suggesting that GelMA is associated with modest changes in osteogenesis markers. However, these observations are based on endpoint measurements at single time points with three biological replicates per group and do not capture the temporal dynamics of the osteogenic process. Although the results indicate derivative-dependent differences in the osteogenic response, the underlying mechanism cannot be determined from the present data and should be interpreted with caution.

Overall, the results of this study revealed that cellular responses to uncrosslinked gelatin derivatives may differ depending on the type of functionalization and cell type. The distinct cellular response profiles observed across the derivatives indicate that functional group chemistry, rather than the shared gelatin backbone alone, may induce different cellular responses. Indeed, gelatin functionalization may alter the physicochemical properties of the gelatin backbone, which could influence cellular responses [23,68]. The consumption of primary amines reduces the cationic character of gelatin [69], while the norbornene anhydride additionally introduces a free carboxylic acid [10,70], making GelNB potentially the most negatively charged of the three derivatives [71]. As the cell membrane carries a net negative charge, cationic biomaterials generally interact more strongly with cells than anionic ones [72,73], although charge may also affect protein adsorption from the medium [74]. The modest cellular changes observed with GelNB compared to other gelatin derivatives suggest that charge alone may not fully explain the observed differences. A complementary explanation involves the chemical reactivity of the introduced functional groups. The methacrylate group in GelMA is an α,β-unsaturated carbonyl with mild electrophilic character, which may interact with cysteine residues on cellular proteins and contribute to stress-related signaling, potentially upregulating stress response genes such as GSTP1 [64,65,75,76]. The phenol group in GelPH is not inherently electrophilic, but enzymatic oxidation to phenoxyl radical or quinone intermediates may indirectly contribute to oxidative stress [77,78]. The norbornene group in GelNB lacks both electrophilic character and a physiologically accessible oxidation pathway, with thiol reactivity requiring an external photoinitiator that is absent under standard culture conditions [76,79].

Although these findings are informative, this study had several limitations. This study was designed as a comparative evaluation of cellular responses to different gelatin functionalizations under controlled, crosslinking-free conditions, and was not intended to resolve the underlying molecular mechanisms. Accordingly, the mechanistic interpretations discussed are speculative and should be considered directions for future investigations rather than definitive conclusions. In addition, only a single degree of functionalization (~50%) and concentration were examined in this study; however, cellular responses to biomaterials can be concentration- and DoF-dependent [9,10]. Hence, the findings reported here may not fully capture the range of responses possible under different exposure conditions. Future studies exploring a broader range of concentrations and DoF values will help clarify the structure–bioactivity relationships. Furthermore, as mentioned before, chemical functionalization inevitably alters the physicochemical properties of the gelatin backbone, which may also contribute to the observed cellular responses, independent of the functional group identity [23,68]. Characterization of these physicochemical differences in future studies will provide a more complete picture of how gelatin functionalization influences cellular behavior. Together, these considerations suggest that the present findings should be interpreted within the scope of the experimental conditions used and that further work will help build a broader understanding to guide gelatin derivative selection in biomedical applications.

Despite these limitations, our findings provide a basis for selecting gelatin derivatives for 3D cell culture applications. Such subtle but reproducible differences may be particularly relevant in applications involving repeated or prolonged exposure to residual precursor molecules, where small effects can accumulate over time. This consideration is particularly relevant in therapeutic applications, where gelatin derivative-based constructs are intended for implantation and direct interaction with surrounding tissues. If crosslinking is incomplete, residual uncrosslinked polymers may leach from the construct, interact with adjacent cells, or enter the bloodstream [19,20,21,22,23,24,25]. Under these conditions, the cytocompatibility of the soluble uncrosslinked form is an important safety consideration that is often overlooked when materials are evaluated exclusively in their crosslinked hydrogel state. In this context, these results may serve as a useful reference for the rational selection of gelatin-derived materials that elicit minimal adverse cellular responses during and after construct fabrication, thereby supporting their safe use in biomedical applications.

## 5. Conclusions

This study demonstrated that soluble, uncrosslinked GelMA, GelNB, and GelPH showed no overt cytotoxicity under the tested conditions in BALB/3T3, HeLa, UE7T-13, or PC-12 cells. However, subtle and measurable differences in cellular responses were observed, and these differences were strongly cell type-dependent. HeLa and PC-12 cells showed limited responses in most parameters, whereas BALB/3T3 cells exhibited mainly GelMA-associated changes in early mitochondrial activity, proliferation, and morphology. In UE7T-13 cells, GelMA showed the largest deviations from controls across the examined readouts, including early mitochondrial activity, cell area, phenotype-related gene expression, and some osteogenic markers, whereas GelPH showed intermediate responses, and GelNB was largely comparable to untreated or native gelatin-treated controls. These findings suggest that gelatin functionalization can be associated with derivative- and cell type-dependent cellular responses even in the absence of crosslinking stimuli. Because chemical functionalization may alter not only the introduced functional group but also the physicochemical properties of the gelatin backbone, the observed responses should not be attributed to functional group identity alone. Although this study was limited to a single polymer concentration and degree of functionalization, it provides a controlled comparative basis for evaluating residual soluble gelatin derivatives in biomaterial design and safety assessments.

## Data Availability

The original contributions of this study are included in the article and Supplementary Material. Further inquiries can be directed to the corresponding author.

## Supplementary Materials

The following supporting information can be downloaded at https://www.mdpi.com/article/10.3390/biom16060836/s1: Figure S1: UV-Vis spectra of GelPH dialysate during purification; Figure S2: ^1^H NMR spectra of (a) GelMA, (b) GelNB, and (c) GelPH; Table S1: Primer information.

## Abbreviations

The following abbreviations are used in this manuscript:

BALB/3T3	Mouse embryonic fibroblast cell line
CD105	Cluster of differentiation 105 (endoglin)
COL1A1	Collagen type I alpha 1 chain
DoF	Degree of functionalization
ECM	Extracellular matrix
GelMA	Gelatin methacrylate
GelNB	Gelatin norbornene
GelPH	Gelatin phenol
GSTP1	Glutathione S-transferase pi 1
HeLa	Human cervical cancer epithelial cell line
NMR	Nuclear magnetic resonance
OCN	Osteocalcin
PC-12	Rat pheochromocytoma neuronal cell line
qPCR	Quantitative polymerase chain reaction
RGD	Arginine-glycine-aspartate (integrin-binding peptide motif)
RNA	Ribonucleic acid
RUNX2	Runt-related transcription factor 2
SOX2	SRY-box transcription factor 2
TGF-β	Transforming growth factor beta
TNBS	2,4,6-trinitrobenzenesulfonic acid
UE7T-13	Immortalized human bone marrow–derived mesenchymal stem cell line
