# Supplementary material for: Functional Group Chemistry Modulates Cellular Responses to Soluble Gelatin Derivatives Independent of Crosslinking
Source: Biomolecules. 2026 Jun 5;16(6):836. doi: 10.3390/biom16060836 (PMC13297345; doi:10.3390/biom16060836)
Supplement: Supplementary file 1 [file biomolecules-16-00836-s001.zip › biomolecules-4327287-supplementary.pdf]

Supplementary materials

Functional Group Chemistry Modulates Cellular Responses to Soluble Gelatin Derivatives Independent of Crosslinking

*Pekik W. Prasetyaningrum, Shinji Sakai**

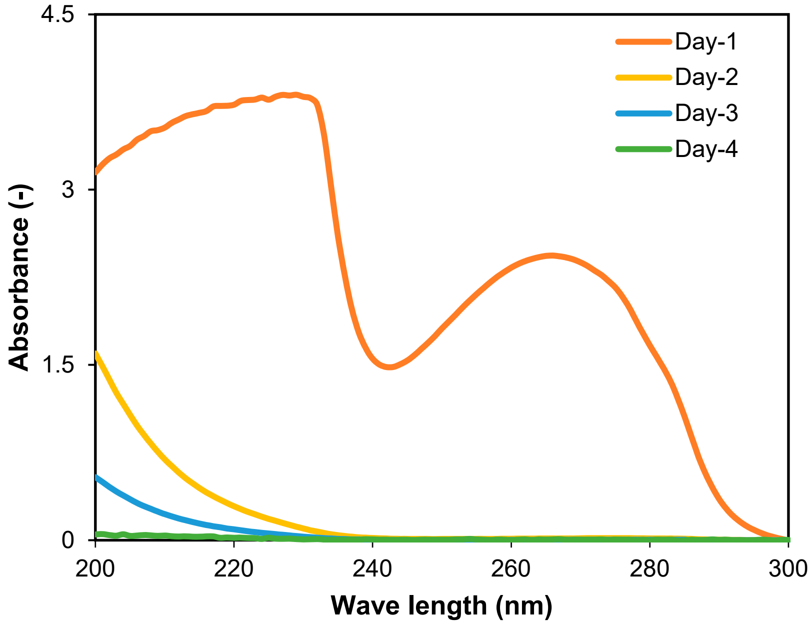

Figure S1. UV–Vis absorbance spectra of the dialysate collected during GelPH purification.

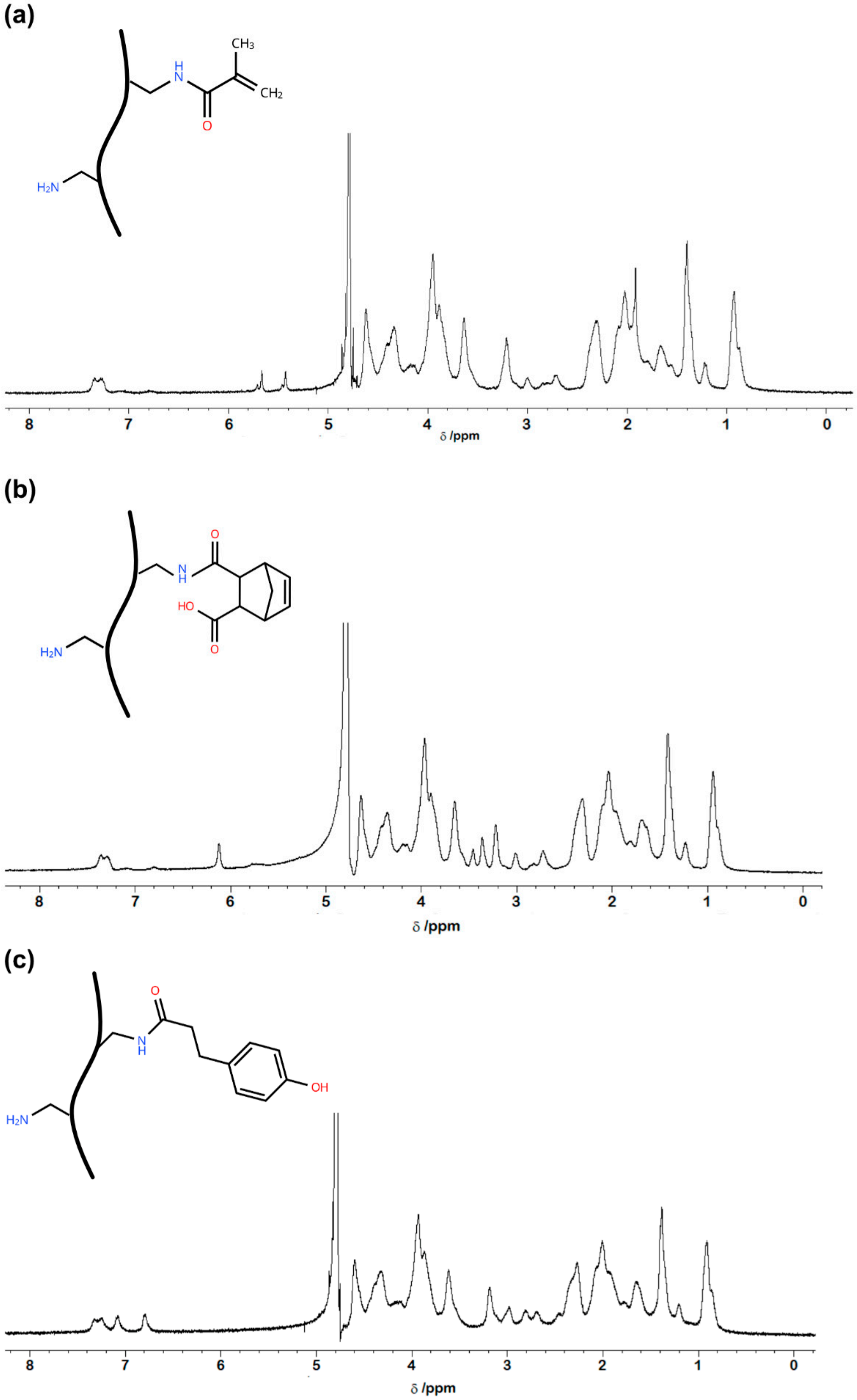

Figure S2. ^{1}H NMR spectra of (a) GelMA, (b) GelNB, and (c) GelPH.

Table S1. Primer information.

**Genes**	**Sequence**
GAPDH (F)	5′-GGAGTCCCTGCCACACTCAG-3′
GAPDH (R)	5′-GGCCCCTCCCCTCTTCA-3′
Vimentin(FW)	5′-TGCCGTTGAAGCTGCTAACTA -3′
Vimentin(RV)	5′-CCAGAGGGAGTGAATCCAGATTA-3′
GSTP1 (Fw)	5′-TGGACATGGTGAATGACGGCGT-3′
GSTP1(Rev)	5′-GGTCTCAAAAGGCTTCAGTTGCC-3′
Endoglin (CD105) (F)	5′-CGGTGGTCAATATCCTGTCGAG-3′
Endoglin (CD105) (R)	5′-AGGAAGTGTGGGCTGAGGTAGA-3′
Sox2 (F)	5′-GCTACAGCATGATGCAGGACCA-3′
Sox2 (R)	5′-TCTGCGAGCTGGTCATGGAGTT-3′
Runx2 (F)	5′-CCCAGTATGAGAGTAGGTGTCC-3′
Runx2 (R)	5′-GGGTAAGACTGGTCATAGGACC-3′
Osteocalcin (F)	5′-CGCTACCTGTATCAATGGCTGG-3′
Osteocalcin (R)	5′-CTCCTGAAAGCCGATGTGGTCA-3′
Col1a1 (F)	5′-GATTCCCTGGACCTAAAGGTGC-3′
Col1a1 (R)	5′-AGCCTCTCCATCTTTGCCAGCA-3′
